# Machine learning of plasma metabolome identifies biomarker panels for metabolic syndrome: findings from the China Suboptimal Health Cohort

**DOI:** 10.1186/s12933-022-01716-0

**Published:** 2022-12-23

**Authors:** Hao Wang, Youxin Wang, Xingang Li, Xuan Deng, Yuanyuan Kong, Wei Wang, Yong Zhou

**Affiliations:** 1 Department of Clinical Epidemiology and Evidence-Based Medicine, Beijing Clinical Research Institute, Beijing Friendship Hospital, Capital Medical University, Beijing, 100050 China; 2 Beijing Key Laboratory of Clinical Epidemiology, School of Public Health, Capital Medical University, Beijing, 100069 China; 3 Center for Precision Medicine, School of Medical and Health Sciences, Edith Cowan University, Perth, WA6027 Australia; 4 Clinical Research Institute, Shanghai General Hospital, Shanghai Jiao Tong University School of Medicine, No. 100 Haining Road, Hongkou District, Shanghai, 200080 China

**Keywords:** Metabolic syndrome, Machine learning, Metabolomics, Biomarkers, Diagnostic models, Amino acid metabolism

## Abstract

**Background:**

Metabolic syndrome (MetS) has been proposed as a clinically identifiable high-risk state for the prediction and prevention of cardiovascular diseases and type 2 diabetes mellitus. As a promising “omics” technology, metabolomics provides an innovative strategy to gain a deeper understanding of the pathophysiology of MetS. The study aimed to systematically investigate the metabolic alterations in MetS and identify biomarker panels for the identification of MetS using machine learning methods.

**Methods:**

Nuclear magnetic resonance-based untargeted metabolomics analysis was performed on 1011 plasma samples (205 MetS patients and 806 healthy controls). Univariate and multivariate analyses were applied to identify metabolic biomarkers for MetS. Metabolic pathway enrichment analysis was performed to reveal the disturbed metabolic pathways related to MetS. Four machine learning algorithms, including support vector machine (SVM), random forest (RF), k-nearest neighbor (KNN), and logistic regression were used to build diagnostic models for MetS.

**Results:**

Thirteen significantly differential metabolites were identified and pathway enrichment revealed that arginine, proline, and glutathione metabolism are disturbed metabolic pathways related to MetS. The protein-metabolite-disease interaction network identified 38 proteins and 23 diseases are associated with 10 MetS-related metabolites. The areas under the receiver operating characteristic curve of the SVM, RF, KNN, and logistic regression models based on metabolic biomarkers were 0.887, 0.993, 0.914, and 0.755, respectively.

**Conclusions:**

The plasma metabolome provides a promising resource of biomarkers for the predictive diagnosis and targeted prevention of MetS. Alterations in amino acid metabolism play significant roles in the pathophysiology of MetS. The biomarker panels and metabolic pathways could be used as preventive targets in dealing with cardiometabolic diseases related to MetS.

**Supplementary Information:**

The online version contains supplementary material available at 10.1186/s12933-022-01716-0.

## Background

Metabolic syndrome (MetS) is a combination of cardiometabolic risk determinants, including central obesity, elevated blood pressure, hyperglycemia, and dyslipidemia [1]. It is a clinically identifiable high-risk state, and MetS patients are at high risk for developing cardiovascular diseases (CVD) and type 2 diabetes mellitus (T2DM) in the future [2]. Depending on the International Diabetes Federation (IDF) definition of MetS, the prevalence of MetS is approximately 25% of all adults in the world [3]. MetS and its consequent chronic diseases lead to high morbidity and mortality rates. In 2016, CVD resulted in 17.9 million deaths [4], and 6.7 million individuals died from T2DM in 2021 worldwide [5]. As these cardiometabolic diseases are among the leading causes of death worldwide, MetS is still a global health issue.

MetS has a multifaceted etiology, involving complex interactions between genetic and environmental factors [6]. The pathophysiological mechanism of MetS is characterized by abnormal metabolism, including dysregulation of glucose and lipid metabolism [7], storage of adipose tissue [8], and chronic low-grade inflammation [9]. Although increasing evidence has shown that insulin resistance and obesity play essential roles in the pathophysiology of MetS [10, 11], several other factors such as increase in cellular oxidative stress [12], low mitochondrial function [13], and dysregulation of the hypothalamic—pituitary—adrenal [14] can also be involved in its pathogenesis. Considering the multi-factorial pathophysiology of MetS, it is inevitable to understand and study the disease from a systemic point of view.

To comprehensively investigate the metabolic characterization of MetS and its role in the development of consequent cardiometabolic diseases, several attempts have been made to screen biomarkers using various omics technologies, including metabolomics [15]. Metabolomics, an emerging “omics” technology, is the profiling of metabolites in a biological system [16]. With the help of metabolomics, the pathophysiological characteristics of MetS have been further explored by looking for potential metabolic biomarkers that provide strong support for the diagnosis and treatment of MetS. These new metabolic insights could lead to a paradigm shift in how preventive interventions and treatment targets are being discovered [17]. In recent years, studies have identified several MetS related metabolic pathways, including amino acid metabolism, glutathione production, gluconeogenesis, and tricarboxylic acid cycle in American, Japanese, and Dutch cohorts [18–20]. However, to the best of our knowledge, the plasma metabolome of MetS patients has not been systematically profiled in a large Chinese cohort to identify biomarkers for the diagnosis of MetS.

The analysis of metabolomics big data is complicated due to its complex structure, such as high dimensionality, high noise levels, and missing values. Conventional statistics-based models are usually not suitable for the analysis of metabolomics big data. Therefore, machine learning methods have become popular for the analysis of metabolomics data, especially for the construction of prediction models based on potential biomarkers for the diagnosis of diseases [21]. Notably, the selection and optimization of machine learning algorithms are also crucial in the diagnosis of diseases.

Taking into account these necessities, the aim of the present study was to comprehensively investigate the plasma metabolic characteristics of MetS in a large well-established Chinese cohort—China Suboptimal Health Cohort Study (COACS), and to screen potential metabolic biomarkers for MetS using proton nuclear magnetic resonance (^1^H-NMR)-based untargeted metabolome profiling. Univariate analysis and multivariate analysis were applied to identify potential metabolic biomarkers for the diagnosis of MetS. Metabolic pathway enrichment analysis was performed to discover which metabolic pathways and metabolites are crucial to the physiopathology of MetS. Four machine learning algorithms, including support vector machine (SVM), random forest (RF), k-nearest neighbor (KNN), and logistic regression were used to build diagnostic models for MetS based on potential metabolic biomarkers. The protein-metabolite-disease interaction network was also explored, so that novel insights or hypotheses regarding the progression of MetS towards its consequent cardiometabolic diseases might be obtained.

## Materials and methods

### Study design and participants

A community-based study was conducted in a Chinese population who received routine health check-ups at the *Jidong* Oilfield Staff Hospital from September 2013 to June 2014. The present study was based on a well-designed cohort named the COACS cohort, which was described previously [22]. All participants were required to meet the following inclusion criteria: (1) aged 18 to 65 years old; and (2) signed informed consent before participation. Participants were excluded if they currently suffering from one or more of the following diseases: (1) diabetes; (2) hypertension; (3) hyperlipemia; (4) cardiovascular or cerebrovascular conditions; (5) cancers; or (6) gout. All participants included in this study signed written informed consent forms. The study was approved by the Ethics Committee of the *Jidong* Oilfield Staff Hospital. Ethnics approval was given in compliance with the Declaration of Helsinki.

### Measurements and sample collection

The demographic characteristics of participants, anthropometric measurements, and biochemical tests were collected as described in our previous study [22]. According to the IDF definition of MetS [23], the participants to be defined as having MetS must have abdominal obesity and any two of the following four phenotypes: (1) systolic blood pressure (SBP) ≥ 130 mmHg and/or diastolic blood pressure (DBP) ≥ 85 mmHg; (2) triglycerides (TG) ≥ 1.7 mmol/L; (3) fasting plasma glucose (FPG) ≥ 5.6 mmol/L; or (4) high-density lipoprotein cholesterol (HDL-C) < 1.03 mmol/L in men or < 1.29 mmol/L in women. Abdominal obesity was defined as waist circumference (WC) ≥ 90 cm in men and WC ≥ 80 cm in women [23]. After at least a 12-h fasting, blood samples were collected from all participants using venipuncture in the morning. The plasma samples were separated in the laboratory after centrifugation at 4 °C, for 10 min at 3000 × g. Then, the samples were stored at − 80 °C immediately, and freeze–thaw cycles were strictly avoided until metabolomic analysis [22].

### Untargeted ^1^H-NMR metabolomics analysis

Plasma samples were thawed at 4 °C. Once thawed, 200 μL of plasma was added to 400 μL of 0.045 M phosphate-buffered saline (PBS) prepared in deuterium oxide (D_2_O) and vortexed for 10 s. The mixture was centrifuged at 13,000 rpm for 15 min at 4 °C. Then 550 μL of supernatant was transferred into 5 mm NMR tubes for further analyses.

All ^1^H-NMR spectra of plasma samples were acquired using a Varian VNMRS 600 MHz spectrometer (Agilent Technologies, USA) operating at a ^1^H frequency of 599.77 MHz. One-domensional (1D) ^1^H-NMR spectra were recorded using the Carr-Purcell-Meiboom-Gill (CPMG) pulse sequence. Each spectrum was acquired with 128 scans per sample using a spectral window of 16.4 ppm. The temperature was kept constant at 25 °C. Water suppression was achieved by using gated irradiation focused on the water frequency. All raw spectra files were obtained using VnmrJ software (Agilent Technologies, USA).

### Data analysis and statistics

**Fig. 1:**
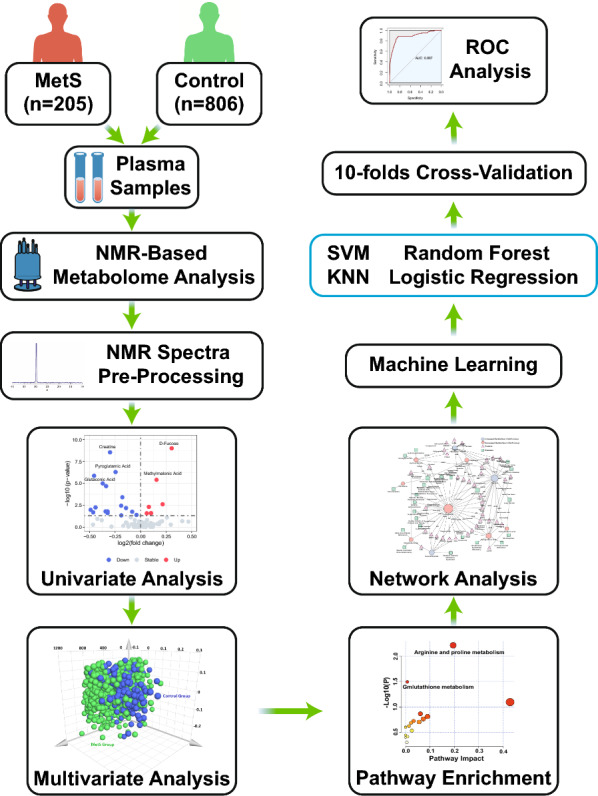
Study design and data analysis workflow MetS, metabolic syndrome; NMR, nuclear magnetic resonance; ROC, receiver operating characteristic; SVM, support vector machine; KNN, k-nearest neighbor

The study design and data analysis workflow are shown in Fig. 1. The raw NMR data were recorded in the form of free induction decay (FID) files which are time-domain spectra. Then the FID files were Fourier transformed into frequency domain spectra using NMRProcFlow software [24]. To remove effects of possible variations on the water suppression efficiency, the region of the water signal was discarded. NMRProcFlow was applied for the preprocessing of NMR spectra data, including phase correction, baseline correction, chemical shift referencing, and spectra alignment [24]. After the constant sum normalization of the spectra, the data matrix was exported to the ASICS R package for the identification and quantification of metabolites. ASICS is based on a library of pure metabolite spectra that is used as a reference to fit a unpenalized model followed by the control of the family wise error rate (FWER). Then the model fit provides the relative quantifications of metabolites in each sample [25].

The data are presented as the means and standard deviations (SDs) if the continuous variables conformed to normal distribution. Otherwise, medians and interquartile ranges (IQRs) were used in descriptive statistics. The differences in continuous variables between the MetS and control groups were tested by *Student t*-test or *Wilcoxon* rank-sum test. Categorical variables are represented as frequencies and percentages. The *Chi-square* test or *Fisher’s* exact test was used to examine the differences in categorical variables between the two groups. The multiple testing corrections were controlled by using the false discovery rate (FDR).

The orthogonal partial least squares projection-discriminant analysis (OPLS-DA) model was performed to identify the metabolic biomarkers using SIMCA, version 14.1 (Umetrics, Umea, Sweden). To estimate the association between metabolic biomarkers and cardiometabolic risk factors, *Spearman’s* rank correlation was performed and visualized using the “*corrplot*” R package. Metabolic pathway analysis and protein-metabolite-disease interaction network analysis were performed by using MetaboAnalyst [26], and Cytoscape, version 3.7.1 (National Institute of General Medical Sciences, Bethesda, USA) was used to create the interaction networks. The diagnostic models for MetS were constructed by using 4 machine learning algorithms, including SVM (“*e1071*” R package), RF (“*randomForest*” R package), KNN ( “*kknn*” R package), and logistic regression (“*glm*” R package). The receiver operating characteristic (ROC) curves were used to evaluate the predictive performance of the models. The area under the curve (AUC) and 95% bootstrap confidence intervals (CI) were also estimated.

Statistical analyses were performed using R, version 4.1.2 (R Foundation for Statistical Computing) and SPSS 25.0 (IBM Corporation, New York, USA). Two-tailed* P* < 0.05 was considered statistically significant.

## Results

### Clinical characteristics of the study population

In total, 205 MetS patients and 806 healthy controls were analysed in the present study. The average ages of the MetS and control groups were 57.21 ± 10.00 and 47.05 ± 12.93 years, respectively. The levels of body mass index (BMI), SBP, DBP, hip circumference (HC), WC, waist-to-hip ratio (WHR), FPG, TG, total cholesterol (TC), low-density lipoprotein cholesterol (LDL-C), blood urea nitrogen (BUN), and creatinine (Cr) were significantly higher in the MetS group than those in the control group, whereas a significantly lower level of HDL-C was observed in the MetS group (all *P* < 0.05). Aside from these, significantly different frequencies of abdominal obesity, elevated blood pressure, elevated FPG, elevated TG, and reduced HDL-C phenotypes were observed between the two groups (all *P* < 0.05). The details about the demographic, biochemical, and anthropometric characteristics of the MetS patients and healthy controls are presented in Table 1.

**Table 1:** Characteristics of the study participants

Characteristics	MetS group (N = 205)	Control group (N = 806)	*P* value
Age (years)	57.21 ± 10.00	47.05 ± 12.93	< 0.001
Male (%)	97 (47.32)	331 (41.07)	0.106
SBP (mmHg)	141.06 ± 19.90	118.79 ± 14.39	< 0.001
DBP (mmHg)	86.04 ± 12.83	75.30 ± 9.51	< 0.001
BMI (kg/m^2^)	26.99 ± 3.01	23.14 ± 3.06	< 0.001
WC (cm)	94.34 ± 8.14	81.76 ± 9.03	< 0.001
HC (cm)	102.54 ± 6.58	95.81 ± 7.16	< 0.001
WHR	0.92 ± 0.06	0.85 ± 0.07	< 0.001
FPG (mmol/L)	6.12 ± 1.66	5.06 ± 0.75	< 0.001
TC (mmol/L)	4.75 ± 1.01	4.31 ± 0.72	< 0.001
TG (mmol/L)	1.89 ± 1.24	1.01 ± 0.41	< 0.001
HDL-C (mmol/L)	1.08 ± 0.19	1.29 ± 0.27	< 0.001
LDL-C (mmol/L)	2.77 ± 0.71	2.36 ± 0.51	< 0.001
BUN (mmol/L)	5.21 ± 1.59	4.83 ± 1.43	0.001
Cr (μmol/L)	76.89 ± 14.83	74.54 ± 12.75	0.035
Abdominal obesity (%)	205 (100.00)	305 (37.84)	< 0.001
Raised BP (%)	170 (82.93)	179 (22.21)	< 0.001
Raised FPG (%)	127 (61.95)	86 (10.67)	< 0.001
Raised TG (%)	92 (44.88)	29 (3.60)	< 0.001
Reduced HDL-C (%)	152 (74.15)	271 (33.62)	< 0.001

Data are presented as means ± SDs or frequencies (percentages)

*MetS* metabolic syndrome, *SD* standard deviation, *SBP* systolic blood pressure, *DBP* diastolic blood pressure, *BP* blood pressure, *BMI* body mass index, *WC* waist circumference, *HC* hip circumference, *WHR* waist-to-hip ratio, *FPG* fasting plasma glucose, *TC* total cholesterol, *TG* triglycerides, *HDL-C* high-density lipoprotein cholesterol, *LDL-C* low-density lipoprotein cholesterol, *BUN* blood urea nitrogen, *Cr* creatinine

*P* < 0.05 is considered statistically significant

### Identification of metabolic biomarkers

**Fig. 2:**
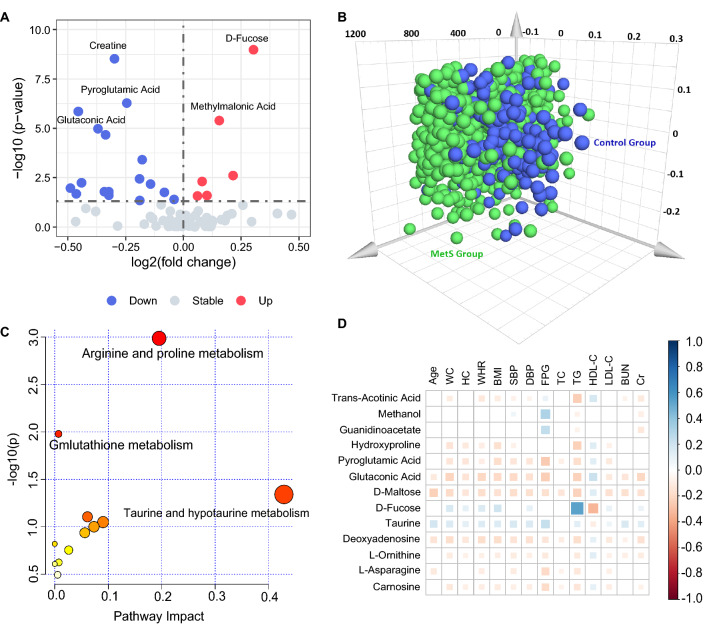
Identification of metabolic biomarkers and disturbed pathways related to metabolic syndrome. **A** Volcano plot of candidate metabolic biomarkers. **B** Orthogonal projection to latent structure-discriminant analysis (OPLS-DS) score plots. **C** Disturbed metabolic pathways in MetS individuals. **D** Correlation coefficient matrix between 13 potential metabolic biomarkers and 14 cardiometabolic risk factors. Statistically significant correlations between two metabolites are shown, while the insignificant correlation coefficients are blank in the boxes. The positive correlations are represented by blue color, while negative correlations are represented by red color; WC, waist circumference; HC, hip circumference; WHR, Waist-to-hip ratio; BMI, body mass index; SBP, systolic blood pressure; DBP, diastolic blood pressure; TC, total cholesterol; TG, triglycerides; HDL-C, high-density lipoprotein cholesterol; LDL-C, low-density lipoprotein cholesterol; BUN, blood urea nitrogen; Cr, creatinine. *P* < 0.05 is considered statistically significant. The detailed correlation coefficients and *P* values were shown in Additional file 4

The metabolome of 1011 plasma samples was analysed using ^1^H-NMR, and the stacked NMR spectra are shown in Additional file 1. After the preprocessing of NMR spectra, identification and quantification of metabolites, and removal of missing values, 85 metabolites were identified successfully (Fig. 2A and Additional file 2). The variable importance on projection (VIP) values of each metabolite was calculated by the OPLS-DA model, and the metabolites with VIP values > 1 were considered the potential candidate metabolites.The number of latent variables in the OPLS-DA model was chosen according to cross-validation. The cumulative R^2^Y and cumulative Q^2^ values of the OPLS-DA model were calculated to estimate the “goodness of fit” and the predictive ability of the model. The OPLS-DA model yielded a cumulative R^2^Y of 0.207 and a cumulative Q^2^ of 0.161. The OPLS-DA score plot showed that the MetS patients were separated from the healthy controls (Fig. 2B). Among the 85 candidate metabolites, 13 metabolites with VIP values > 1, *P* values < 0.05, and FDR-, age-adjusted *P* values < 0.05 were identified as candidate biomarkers for MetS (Table 2 and Additional file 2).

**Table 2:** Differential metabolites identified between MetS participants and controls

Metabolites	MetS group		Control group		Trend	FC	VIP	*P* value	P* value	*P*^*#*^ value
	Mean	SD	Mean	SD						
Trans-Acotinic Acid	0.0043	0.0031	0.0054	0.0022	Down	0.7917	4.4239	3.543E–03	2.515E–06	2.138E–05
Methanol	0.0051	0.0012	0.0049	0.0008	Up	1.0432	1.0652	3.836E–04	9.162E–03	2.685E–02
Guanidinoacetate	0.0039	0.0011	0.0036	0.0007	Up	1.0579	1.0384	5.379E–04	9.941E–04	4.970E–03
Hydroxyproline	0.0027	0.0020	0.0035	0.0016	Down	0.7738	3.4554	2.930E–06	1.113E–06	1.052E–05
Pyroglutamic Acid	0.0021	0.0009	0.0025	0.0009	Down	0.8438	2.0394	1.155E–11	2.459E–08	5.225E–07
Glutaconic Acid	0.0008	0.0010	0.0014	0.0010	Down	0.5850	2.6766	3.108E–15	4.362E–10	1.236E–08
D-Maltose	0.0014	0.0011	0.0019	0.0008	Down	0.7296	2.2688	1.547E–09	8.209E–08	1.395E–06
D-Fucose	0.0010	0.0005	0.0008	0.0003	Up	1.2343	1.2169	3.648E–10	1.209E–11	1.028E–09
Taurine	0.0017	0.0006	0.0014	0.0005	Up	1.1609	1.5231	1.675E–08	4.260E–04	2.481E–03
Deoxyadenosine	0.0002	0.0004	0.0004	0.0004	Down	0.6409	1.0993	2.990E-06	4.379E–04	2.481E–03
L-Ornithine	0.0010	0.0013	0.0013	0.0013	Down	0.7367	1.8037	3.846E–04	1.291E–03	5.777E–03
L-Asparagine	0.0007	0.0006	0.0009	0.0006	Down	0.7888	1.0710	2.398E–06	4.334E–03	1.635E–02
Carnosine	0.0003	0.0004	0.0004	0.0005	Down	0.6662	1.0302	1.743E–05	3.734E–04	2.442E–03

Down trend, relatively lower levels of metabolites present in MetS group. Up trend, relatively higher levels of metabolites present in the MetS group

*MetS* metabolic syndrome, *FC* fold change, *VIP* variable importance on projection

*P* value, *P* value from Wilcoxon test without adjustment

*P* < 0.05 was considered statistically significant

P^*^ value, *P* value adjusted for age

*P*^#^ Value, *P* value adjusted for age and false discovery rate using the *Benjamini‒Hochberg* method

### Metabolic pathway enrichment analysis

Metabolic pathway analysis was performed to reveal the disturbed metabolic pathways related to MetS based on potential metabolic biomarkers. These metabolites were involved in 12 metabolic pathways (Fig. 2C and Additional file 3). Among these 12 metabolic pathways, two pathways with *P* values < 0.05 and impact values > 0.00 were identified as arginine and proline metabolism, and glutathione metabolism pathways, respectively. The arginine and proline metabolism pathway included 38 metabolites in total, while 3 metabolites (guanidinoacetate, hydroxyproline, and L-ornithine) were measured in this study. The glutathione metabolism pathway included 28 metabolites in total, while 2 metabolites (pyroglutamic acid and L-ornithine) were measured in the present study (Fig. 2C and Additional file 3).

### Association between metabolic biomarkers and cardiometabolic risk factors

**Fig. 3:**
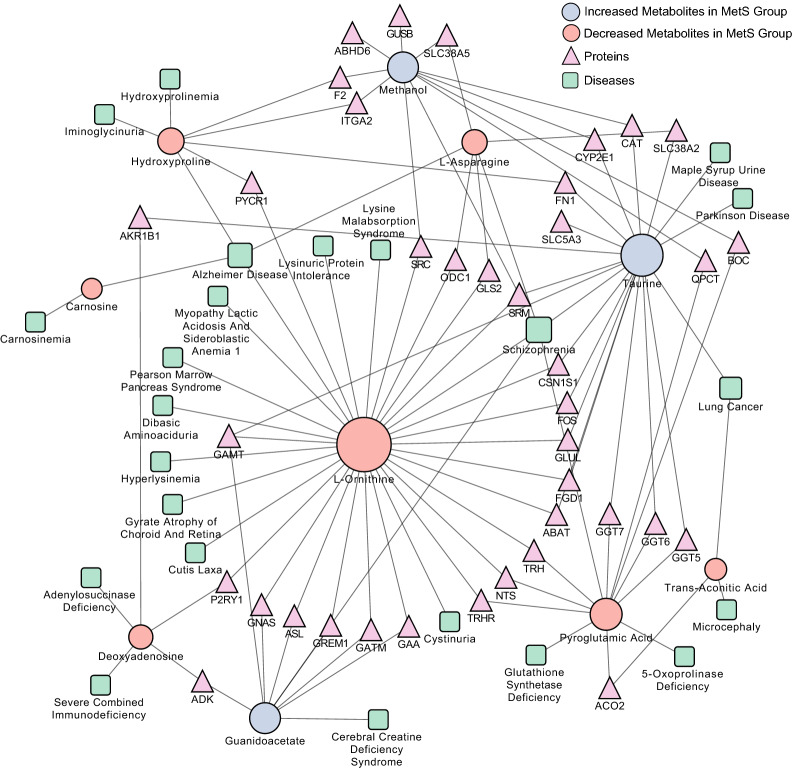
Protein-metabolic-disease network analysis of potential biomarkers for metabolic syndrome

To investigate the potential relationships between 13 metabolic biomarkers and 14 cardiometabolic risk factors, *Spearman’s* correlation coefficients were calculated (Additional file 4). The matrix of correlation coefficients is visualized in Fig. 3. Among the 13 metabolic biomarkers, 13 metabolites were significantly associated with TG, and 10 metabolites were associated with WC, WHR, SBP, FPG, HDL-C, LDL-C, and Cr, followed by 9 metabolites were associated with HC, BMI, and DBP, 6 metabolites were associated with BUN, and 5 metabolites were associated with age and TC (Additional file 4). The significant correlation coefficients ranged from − 0.335 to 0.534. D-Fucose showed the highest correlation with the cardiometabolic risk factors, associated with 9 of the 13 metabolic risk factors. The correlation coefficient between D-fucose and TG was highest (*r* = 0.534, *P* value < 0.001). There were five metabolites correlated with age (*P* values < 0.05), and the correlation coefficients ranged from − 0.238 to 0.188, which were relatively low. D-Maltose and Deoxyadenosine were associated with all the 14 cardiometabolic risk factors included in this study (Fig. 2D and Additional file 4).

### Protein-metabolite-disease interaction network

A protein-metabolite-disease interaction network was constructed to provide a comprehensive understanding of potential functional relationships among potential metabolic biomarkers, proteins, and diseases. Based on the previous knowledge of literature associations, biological pathways, similar structures, and similar functions, the interactions between metabolites and proteins were searched from the Search Tool for Interactions of Chemicals (STITCH) database [27]. There were 38 proteins associated with 10 metabolic biomarkers for MetS (Fig. 3). According to the association between metabolites and diseases in the Human Metabolome Database (HMDB) [28], the metabolite-disease interaction network was also constructed to explore the association between MetS-related metabolites and chronic diseases. Finally, 23 diseases were associated with 10 MetS-related metabolites (Fig. 3).

### Diagnostic models for MetS using machine learning algorithms

**Fig. 4:**
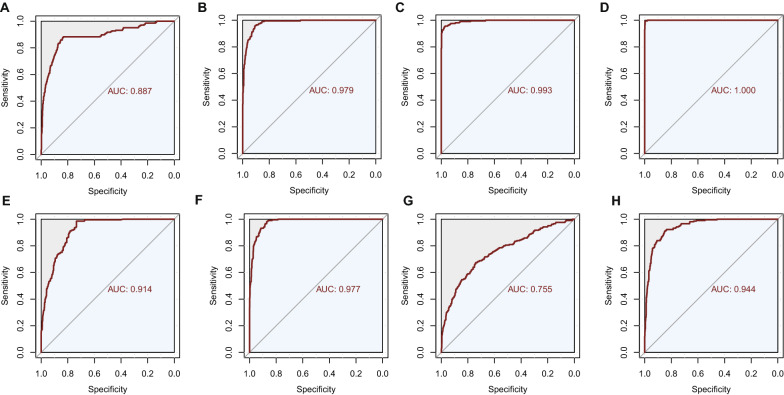
Area under the receiver operating characteristic curves of four machine learning algorithms **A** support vector machine model based on metabolic biomarkers; **B** support vector machine model based on cardiometabolic risk factors; **C** random forest model based on metabolic biomarkers; **D** random forest model based on cardiometabolic risk factors; **E** k-nearest neighbor model based on metabolic biomarkers; **F** k-nearest neighbor model based on cardiometabolic risk factors; **G** logistic regression model based on metabolic biomarkers. **H** logistic regression model based on cardiometabolic risk factors;

After comprehensively profiling the metabolic biomarkers, four machine learning algorithms, including SVM, RF, KNN, and logistic regression, were performed to construct diagnostic models based on 13 metabolic biomarkers. The parameters of different models were tuned using ten-fold cross-validation on the whole dataset. Then, the parameters were applied to the whole dataset to provide final metrics of the suitability of the models for classifying individuals with MetS and healthy controls. Eventually, the kernel used in the SVM model was the radial kernel. The number of trees in the RF model was 500. The number of neighbours in the KNN model was 19. Then the diagnostic models based on 14 cardiometabolic risk factors were also built to compare the predictive ability with models based on metabolic biomarkers. The diagnostic performance of these eight models was shown in Table 3 and Fig. 4, and the AUCs ranged from 0.755 to 0.993 (Fig. 4).

**Table 3:** Diagnostic performance of four machine learning algorithms

Machine learning algorithms	Models	Sensitivity	Specificity	Non-error Rate	AUC (95% CI)
Support vector machine	Metabolites	0.878	0.837	0.846	0.887 (0.857–0.915)
	Risk factors	0.956	0.909	0.919	0.979 (0.971–0.986)
Random forest	Metabolites	0.956	0.971	0.968	0.993 (0.988–0.997)
	Risk factors	0.995	0.996	0.996	1.000 (0.999–1.000)
K-nearest neighbor	Metabolites	0.985	0.734	0.785	0.914 (0.895–0.930)
	Risk factors	0.980	0.871	0.893	0.977 (0.970–0.984)
Logistic regression	Metabolites	0.673	0.742	0.728	0.755 (0.715–0.794)
	Risk factors	0.847	0.912	0.861	0.944 (0.929–0.959)

The 95% CIs of AUCs were estimated using bootstrap resampling for 2000 times

*AUC* area under curve, *CI* confidence interval

## Discussion

Identifying key metabolic biomarkers and pathways relevant to MetS and its progression towards cardiometabolic diseases is considered a viable strategy for the predictive diagnosis and targeted prevention of cardiometabolic diseases. In the present study, we comprehensively described the metabolomic biosignatures of MetS, and the metabolic biosignatures revealed significant differences between MetS patients and healthy participants. Based on the 13 potential metabolic biomarkers for MetS, the pathway analysis suggested that arginine and proline metabolism, and glutathione metabolism pathways were disturbed in MetS patients. Four machine learning algorithms, including SVM, RF, KNN, and logistic regression were used to build diagnostic models for MetS. ROC curve analysis showed that the AUCs of four models based on metabolic biomarkers ranged from 0.755 to 0.993. To our knowledge, the present study is the first to comprehensively provide metabolomic biosignatures of MetS based on a large well-established Chinese cohort by using ^1^H-NMR-based metabolome profiling. Our findings unveiled that metabolome provides a valuable resource of biomarkers for the diagnosis and prevention of MetS and its consequent cardiometabolic diseases. These metabolomic biomarkers also provide a better insight into the critical metabolic pathways associated with MetS and a deeper understanding of its progression towards cardiometabolic diseases. Thus, the MetS-related metabolites and the metabolic patterns of metabolites can be used as potential diagnostic models for population risk stratification and targeted intervention of MetS towards chronic diseases, including CVD and T2DM.

We identified significant differences between MetS patients and healthy controls in cardiovascular risk factors, including BMI, SBP, DBP, HC, WC, WHR, FPG, TG, TC, HDL-C, LDL-C, BUN, and Cr (Table 1). We additionally found that 13 metabolic biomarkers for MetS were also significantly correlated with these cardiovascular risk factors (Fig. 2D). These metabolites may also be affected by these clinical risk factors. Considering that MetS is a constellation of closely related cardiometabolic risk factors, these candidate metabolic biomarkers for MetS could also be potential biomarkers for abdominal obesity, hypertension, hyperglycemia and dyslipidemia. Plasma concentrations of these metabolites may be important indicators of the pathophysiological mechanism of MetS and provide insights into effective treatments for cardiometabolic risk factors.

Pathway analysis revealed that the arginine and proline metabolism pathways are associated with MetS. Guanidinoacetate, hydroxyproline, and L-ornithine are the measured metabolites that participate in arginine and proline metabolism. Arginine, a semi-essential amino acid, is one of the most metabolically versatile amino acids. It serves as a precursor for the synthesis of urea, polyamines, proline, nitric oxide, creatine, glutamate, and agmatine [29]. Numerous studies have suggested that intravenous use or dietary supplementation of arginine is beneficial in improving cardiovascular, pulmonary, renal, gastrointestinal, liver, and immune functions, as well as enhancing insulin sensitivity and maintaining tissue integrity [30]. The dynamic balance of L-arginine may be an endogenous determinant of arterial tone in hypertension [31]. Mirmiran et al. [32] found that plant-derived L-arginine could be a potentially protective factor against the development of MetS and its phenotypes, and higher intakes of animal-derived L-arginine could be a dietary risk factor for the development of MetS. The potential modulatory effects of L-arginine supplementation are currently considered a novel and effective strategy for the treatment and prevention of MetS and its phenotypes, including central obesity, hyperglycemia, and dyslipidemia [33, 34]. In our study, a significantly higher level of guanidinoacetate was found in MetS patients. Otherwise, a significantly lower-level of L-ornithine was found in MetS patients. These findings supported that MetS and its phenotypes are associated with the imbalance of arginine metabolism, and these biomarkers can be used as new intervention targets for MetS and cardiometabolic risk factors.

Hydroxyproline, a nonessential amino acid, is a structurally and physiologically important amino acid in humans. Emerging evidence proves that the oxidation of hydroxyproline plays a significant role in regulating oxidative defense, apoptosis, and angiogenesis [35]. Studies have suggested that chronic low-grade inflammation and oxidative stress in obese individuals are the important underlying mechanism that led to the development of MetS through changed cellular and nuclear mechanisms, including impairments in DNA damage reparation and cell cycle regulation [12]. Capel et al. [36] observed that metabolites from arginine and proline metabolism pathways were significantly different between MetS patients and healthy controls. Targeted and untargeted metabolite profiling found that hydroxyproline could be a potential metabolic biomarker for cardiovascular diseases [37]. In the present study, the significantly lower level of hydroxyproline in MetS patients showed that plasma hydroxyproline was associated with MetS and its phenotypes of the cardiovascular system. The findings of the present study indicated that plasma hydroxyproline could be used as a potential biomarker for the progression of MetS towards cardiovascular diseases, and hydroxyproline metabolism could serve as treatment targets for MetS and cardiometabolic diseases.

Pyroglutamic acid and L-ornithine are the measured metabolites that participate in glutathione metabolism. Glutathione is a low-molecular-weight tripeptide composed of the amino acid glutamine, cysteine, and glycine [38]. It plays a pivotal role in maintaining redox balance, reducing oxidative stress, enhancing metabolic detoxification, and regulating the immune response [38]. A great body of evidence suggested that glutathione may be a potential biomarker and treatment target in various chronic, metabolic diseases, such as hypertension, T2DM, and CVD [39–41]. Sekhar et al. [42] found that patients with uncontrolled T2DM have severely decreased synthesis of glutathione. In the present study, significantly lower levels of pyroglutamic acid and L-ornithine in the glutathione metabolism pathway were observed in MetS patients. These findings showed that deficient synthesis of glutathione occurred in MetS patients, which indicated that elevated oxidative stress may play a significant role in the pathophysiology of MetS.

The metabolite-protein interaction network enables the visualization and exploration of interactions between metabolites and functionally related proteins. This visual network can be used to acquire innovative insights into the pathophysiology of MetS and its progression towards cardiometabolic diseases. According to the association between metabolites and diseases obtained from the HMDB database, a metabolite-disease interaction network was also produced to explore the disease-related metabolites. In the present study, MetS-related metabolic biomarkers were found to be associated with 23 diseases, such as Parkinson’s disease, Alzheimer’s disease, lung cancer, and schizophrenia. Some of these diseases were reported to be associated with MetS. The lower levels of L-ornithine, hydroxyproline, carnosine, and L-asparagine were observed in the individuals with MetS. All these four potential metabolic biomarkers for MetS were also found to be associated with Alzheimer's disease. Previous studies supported that MetS and T2DM are risk factors for Alzheimer's disease [43]. The underlying mechanism of MetS toward Alzheimer's disease may be involved in the aberrations in the amino acid metabolism in MetS patients.

Several limitations in the present study need to be addressed. Firstly, the causal effect was difficult to infer in data from a cross-sectional study design. The observed MetS-related metabolites may be the consequences rather than causes of MetS and its phenotypes. To investigate the causations between metabolic biomarkers and cardiometabolic risk factors, Mendelian randomization studies in the same cohort of participants are also needed. Secondly, given the semi-quantitative nature of untargeted metabolomics profiling, a targeted metabolomics study is underway against the same cohort to validate the potential biomarkers and pathways based on the findings in the present study. Despite the limitations, the present study has provided a novel strategy that plasma metabolomics offers an innovative alternative for the recognition of MetS. Building on the findings, further studies from diverse populations and geographical areas are warranted.

## Conclusions

The early diagnosis of MetS has the potential to identify the patients who are at high risk of developing CVD and T2DM at early stages, and evidence-based intervention for MetS may be a cost-effective method for targeted prevention, and personalized intervention for cardiometabolic diseases, such as CVD and T2DM. A total of 13 metabolites, including trans-acotinic acid, methanol, guanidinoacetate, hydroxyproline, pyroglutamic acid, glutaconic acid, D-maltose, D-fucose, taurine, deoxyadenosine, L-ornithine, L-asparagine, and carnosine, were selected as candidate biomarkers for MetS. The present study revealed the potential value of metabolomic biomarkers for the predictive diagnosis of MetS. MetS patients have a universal metabolic disturbance. The significantly higher level of guanidinoacetate and significantly lower level of L-ornithine in MetS patients indicated that the disturbance of arginine metabolism plays a significant role in the pathophysiologic mechanism of MetS and its phenotypes. Hydroxyproline and glutathione metabolism also play potential roles in the pathophysiologic mechanism of MetS. These findings determined the potential utility of MetS-related metabolic biomarkers and pathways for targeted prevention and personalized therapy of cardiometabolic diseases.

## Supplementary Information

**Additional file 1: Figure S1**. The stacked NMR spectra of plasma samples from 205 MetS patients and 806 healthy controls.

**Additional file 2: Table S1**. Statistical analysis of identified metabolites.

**Additional file 3: Table S2**. Pathway enrichment analysis of potential metabolic biomarkers.

**Additional file 4: Table S3**. *Spearman*’s correlation coefficients between the 13 metabolomic biomarkers and 14 cardiometabolic risk factors.

## Data Availability

The datasets generated during and analysed during the current study are available from the corresponding author on reasonable request.

## Abbreviations

1D: One-dimensional
1H-NMR: Proton nuclear magnetic resonance
AUC: Area under the curve
BMI: Body mass index
BUN: Blood urea nitrogen
CI: Confidence interval
COACS: China Suboptimal Health Cohort Study
Cr: Creatinine
CVD: Cardiovascular disease
D_2_O: Deuterium oxide
DBP: Diastolic blood pressure
FDR: False discovery rate
FID: Free induction decay
FPG: Fasting plasma glucose
HC: Hip circumference
HDL-C: High-density lipoprotein cholesterol
HMDB: Human metabolome database
IDF: International Diabetes Federation
IQR: Interquartile range
KNN: K-nearest neighbour
LDL-C: Low-density lipoprotein cholesterol
MetS: Metabolic syndrome
OPLS-DA: Orthogonal partial least squares projection-discriminant analysis
PBS: Phosphate-buffered saline
RF: Random forest
ROC: Receiver operating characteristic
SBP: Systolic blood pressure
SD: Standard deviation
STITCH: Search tool for interactions of chemicals
SVM: Support vector machine
T2DM: Type 2 diabetes mellitus
TC: Total cholesterol
TG: Triglycerides
VIP: Variable importance on projection
WC: Waist circumference
WHR: Waist-to-hip ratio

## References

[1] Alberti KG, Zimmet P, Shaw J (2006). Metabolic syndrome—a new world-wide definition a consensus statement from the international diabetes federation. Diabet Med.

[2] Alberti KG, Eckel RH, Grundy SM, Zimmet PZ, Cleeman JI, Donato KA (2009). Harmonizing the metabolic syndrome: a joint interim statement of the international diabetes federation task force on epidemiology and prevention; national heart, lung, and blood institute; american heart association; world heart federation; international atherosclerosis society; and international association for the study of obesity. Circulation.

[3] O'Neill S, O'Driscoll L (2015). Metabolic syndrome: a closer look at the growing epidemic and its associated pathologies. Obes Rev.

[4] World health organization. Global health observatory data 2016 http://www.who.int/gho/en/ Accessed 7 July 2022.

[5] International diabetes federation. international diabetes federation diabetes atlas 10th edition 2017 https://diabetesatlas.org/atlas/tenth-edition/ Accessed 7 July 2022. 10.1016/j.diabres.2021.109083 34883188

[6] Fanning E, O'Shea D (2018). Genetics and the metabolic syndrome. Clin Dermatol.

[7] Despres JP, Lemieux I, Bergeron J, Pibarot P, Mathieu P, Larose E (2008). Abdominal obesity and the metabolic syndrome: contribution to global cardiometabolic risk. Arterioscler Thromb Vasc Biol.

[8] Le Lay S, Dugail I (2009). Connecting lipid droplet biology and the metabolic syndrome. Prog Lipid Res.

[9] Hotamisligil GS (2006). Inflammation and metabolic disorders. Nature.

[10] Romeo GR, Lee J, Shoelson SE (2012). Metabolic syndrome, insulin resistance, and roles of inflammation—mechanisms and therapeutic targets. Arterioscler Thromb Vasc Biol.

[11] Laakso M, Kuusisto J (2014). Insulin resistance and hyperglycaemia in cardiovascular disease development. Nat Rev Endocrinol.

[12] Rani V, Deep G, Singh RK, Palle K, Yadav UC (2016). Oxidative stress and metabolic disorders: pathogenesis and therapeutic strategies. Life Sci.

[13] Bhatti JS, Bhatti GK, Reddy PH (2017). Mitochondrial dysfunction and oxidative stress in metabolic disorders—a step towards mitochondria based therapeutic strategies. Biochim Biophys Acta Mol Basis Dis.

[14] Chrousos GP (2000). The role of stress and the hypothalamic-pituitary-adrenal axis in the pathogenesis of the metabolic syndrome: neuro-endocrine and target tissue-related causes. Int J Obes Relat Metab Disord.

[15] Allam-Ndoul B, Guenard F, Garneau V, Cormier H, Barbier O, Perusse L (2016). Association between metabolite profiles, metabolic syndrome and obesity status. Nutrients.

[16] Fiehn O (2002). Metabolomics—the link between genotypes and phenotypes. Plant MolBiol.

[17] Wishart DS (2016). Emerging applications of metabolomics in drug discovery and precision medicine. Nat Rev Drug Discov.

[18] Roberts JA, Varma VR, Huang CW, An Y, Oommen A, Tanaka T (2020). Blood metabolite signature of metabolic syndrome implicates alterations in amino acid metabolism: findings from the baltimore longitudinal study of aging (BLSA) and the Tsuruoka metabolomics cohort study (TMCS). Int J Mol Sci.

[19] Surowiec I, Noordam R, Bennett K, Beekman M, Slagboom PE, Lundstedt T (2019). Metabolomic and lipidomic assessment of the metabolic syndrome in Dutch middle-aged individuals reveals novel biological signatures separating health and disease. Metabolomics.

[20] Mahajan UV, Varma VR, Huang CW, An Y, Tanaka T, Ferrucci L (2020). Blood metabolite signatures of metabolic syndrome in two cross-cultural older adult cohorts. Int J Mol Sci.

[21] Liebal UW, Phan ANT, Sudhakar M, Raman K, Blank LM (2020). Machine learning applications for mass spectrometry-based metabolomics. Metabolites.

[22] Wang Y, Ge S, Yan Y, Wang A, Zhao Z, Yu X (2016). China suboptimal health cohort study: rationale, design and baseline characteristics. J Transl Med.

[23] International diabetes federation. The IDF consensus worldwide denition of the metabolic syndrome 2005 https://www.idf.org/e-library/consensus-statements/60-idfconsensus-worldwide-definitionof-the-metabolic-syndrome. Accessed 7 July 2022

[24] Jacob D, Deborde C, Lefebvre M, Maucourt M, Moing A (2017). NMRProcFlow: a graphical and interactive tool dedicated to 1D spectra processing for NMR-based metabolomics. Metabolomics.

[25] Tardivel PJC, Canlet C, Lefort G, Tremblay-Franco M, Debrauwer L, Concordet D (2017). ASICS: an automatic method for identification and quantification of metabolites in complex 1D 1H NMR spectra. Metabolomics.

[26] Xia J, Sinelnikov IV, Han B, Wishart DS (2015). Metaboanalyst 3.0—making metabolomics more meaningful. Nucleic Acids Res.

[27] Szklarczyk D, Santos A, von Mering C, Jensen LJ, Bork P, Kuhn M (2016). STITCH 5: augmenting protein-chemical interaction networks with tissue and affinity data. Nucleic Acids Res.

[28] Wishart DS, Feunang YD, Marcu A, Guo AC, Liang K, Vazquez-Fresno R (2018). HMDB 40: the human metabolome database for. Nucleic Acids Res.

[29] Wu G, Morris SM (1998). Arginine metabolism: nitric oxide and beyond. Biochem J.

[30] Wu G, Bazer FW, Davis TA, Kim SW, Li P, Marc Rhoads J (2009). Arginine metabolism and nutrition in growth, health and disease. Amino Acids.

[31] Gokce N (2004). L-arginine and hypertension. J Nutr.

[32] Mirmiran P, Moghadam SK, Bahadoran Z, Ghasemi A, Azizi F (2017). Dietary L-arginine intakes and the risk of metabolic syndrome: a 6-year follow-up in tehran lipid and glucose study. Prev Nutr Food Sci.

[33] Jobgen WS, Fried SK, Fu WJ, Meininger CJ, Wu G (2006). Regulatory role for the arginine-nitric oxide pathway in metabolism of energy substrates. J Nutr Biochem.

[34] Lucotti P, Setola E, Monti LD, Galluccio E, Costa S, Sandoli EP (2006). Beneficial effects of a long-term oral L-arginine treatment added to a hypocaloric diet and exercise training program in obese, insulin-resistant type 2 diabetic patients. Am J Physiol Endocrinol Metab.

[35] Wu Z, Hou Y, Dai Z, Hu CA, Wu G (2019). Metabolism, nutrition, and redox signaling of hydroxyproline. Antioxid Redox Signal.

[36] Capel F, Bongard V, Malpuech-Brugere C, Karoly E, Michelotti GA, Rigaudiere JP (2020). Metabolomics reveals plausible interactive effects between dairy product consumption and metabolic syndrome in humans. Clin Nutr.

[37] Teul J, Garcia A, Tunon J, Martin-Ventura JL, Tarin N, Bescos LL (2011). Targeted and non-targeted metabolic time trajectory in plasma of patients after acute coronary syndrome. J Pharm Biomed Anal.

[38] Pizzorno J (2014). Glutathione!. Integr Med.

[39] Ballatori N, Krance SM, Notenboom S, Shi S, Tieu K, Hammond CL (2009). Glutathione dysregulation and the etiology and progression of human diseases. Biol Chem.

[40] Franco R, Schoneveld OJ, Pappa A, Panayiotidis MI (2007). The central role of glutathione in the pathophysiology of human diseases. Arch Physiol Biochem.

[41] Robaczewska J, Kedziora-Kornatowska K, Kozakiewicz M, Zary-Sikorska E, Pawluk H, Pawliszak W (2016). Role of glutathione metabolism and glutathione-related antioxidant defense systems in hypertension. J Physiol Pharmacol.

[42] Sekhar RV, McKay SV, Patel SG, Guthikonda AP, Reddy VT, Balasubramanyam A (2011). Glutathione synthesis is diminished in patients with uncontrolled diabetes and restored by dietary supplementation with cysteine and glycine. Diabetes Care.

[43] Takechi R, Lam V, Mamo JCL (2022). Diabetic hypertriglyceridaemia and Alzheimer’s disease: causal or not?. Curr Opin Endocrinol Diabetes Obes.

