# Supplementary material for: Machine learning of plasma metabolome identifies biomarker panels for metabolic syndrome: findings from the China Suboptimal Health Cohort
Source: Cardiovasc Diabetol. 2022 Dec 23;21:288. doi: 10.1186/s12933-022-01716-0 (PMC9789589; doi:10.1186/s12933-022-01716-0)
Supplement: Supplementary file 1 — Additional file 1: Figure S1. The stacked NMR spectra of plasma samples from 205 MetS patients and 806 healthy controls. [file 12933_2022_1716_MOESM1_ESM.pdf]

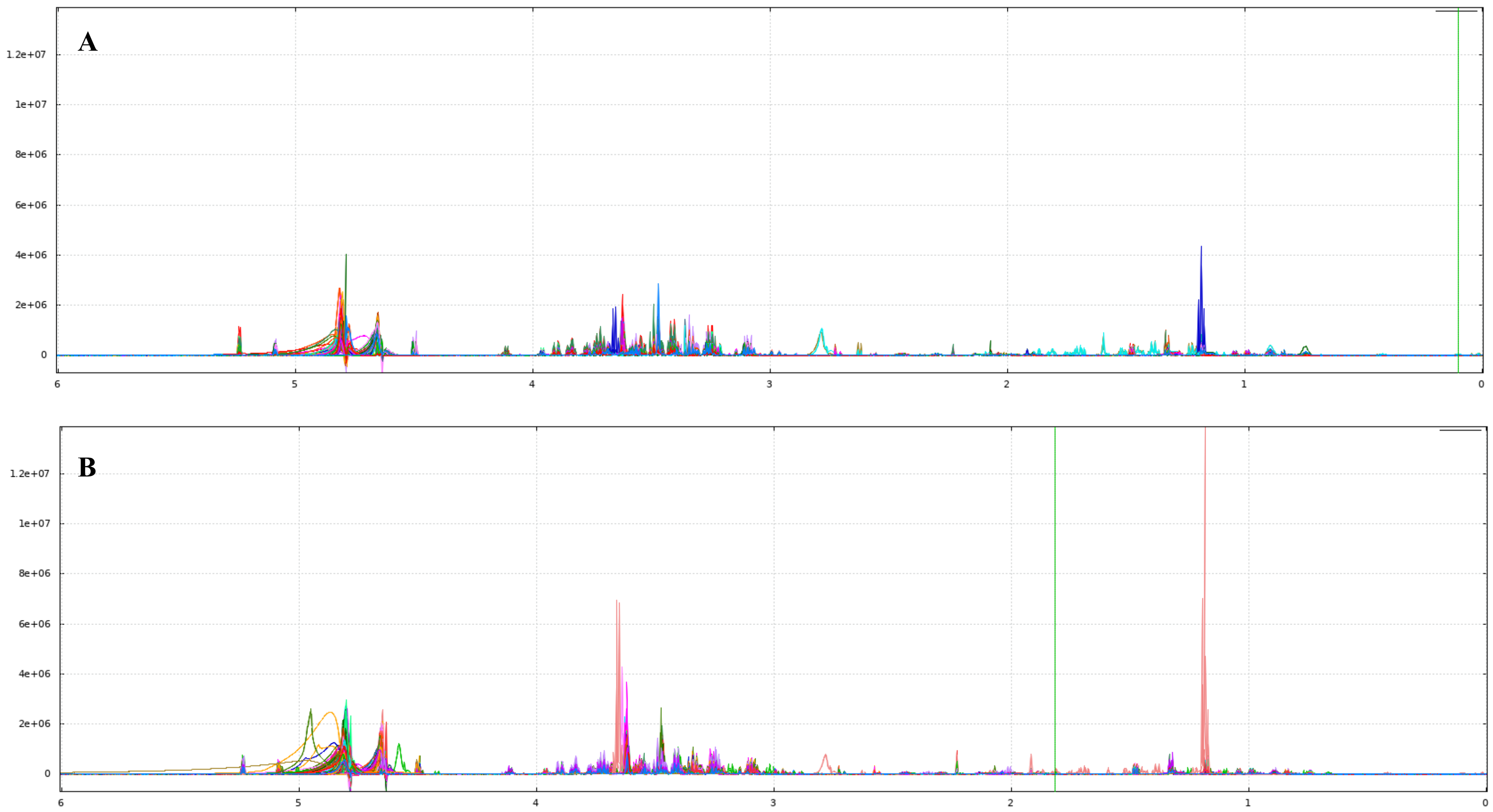

Additional file 1: Figure S1. stacked NMR spectra of plasma samples from 205 MetS patients and 806 healthy controls

Note: NMR, nuclear magnetic resonance; MetS, metabolic syndrome; (A) The stacked NMR spectra of plasma samples from MetS patients; (B) The stacked NMR spectra of plasma samples from healthy controls.
