# Supplementary material for: Machine learning of plasma metabolome identifies biomarker panels for metabolic syndrome: findings from the China Suboptimal Health Cohort
Source: Cardiovasc Diabetol. 2022 Dec 23;21:288. doi: 10.1186/s12933-022-01716-0 (PMC9789589; doi:10.1186/s12933-022-01716-0)
Supplement: Supplementary file 3 — Additional file 3: Table S2. Pathway enrichment analysis of potential metabolic biomarkers. [file 12933_2022_1716_MOESM3_ESM.pdf]

Additional file 3: Table S2. Enrichment pathway analysis of potential metabolic biomarkers

Metabolic pathways	Total metabolites in the pathways, *N*	Observed metabolites, *N*	*P* value	Impact value	Observed metabolites names
Arginine and proline metabolism	38	3	<0.01	0.20	Guanidinoacetate, Hydroxyproline, L-Ornithine
Glutathione metabolism	28	2	0.01	0.01	Pyroglutamic Acid, L-Ornithine
Taurine and hypotaurine metabolism	8	1	0.05	0.43	Taurine
Arginine biosynthesis	14	1	0.08	0.06	L-Ornithine
Histidine metabolism	16	1	0.09	0.09	Carnosine
Starch and sucrose metabolism	18	1	0.10	0.07	D-Maltose
beta-Alanine metabolism	21	1	0.12	0.06	D-Sorbitol
Alanine, aspartate and glutamate metabolism	28	1	0.15	0.00	L-Asparagine
Glycine, serine and threonine metabolism	33	1	0.18	0.03	Guanidinoacetate
Primary bile acid biosynthesis	46	1	0.24	0.01	Taurine
Aminoacyl-tRNA biosynthesis	48	1	0.25	0.00	L-Asparagine
Purine metabolism	65	1	0.32	0.01	Deoxyadenosine

Note: *N*, number of metabolites. *P* Value calculated using *mummichog* method. Impact value, pathway impact value calculated from pathway topology analysis.
